# Influence of the medial longitudinal arch of the foot in adult women in ankle isokinetic performance: a cross-sectional study

**DOI:** 10.1186/s13047-021-00479-3

**Published:** 2021-06-12

**Authors:** Leandro C. Guenka, Aline C. Carrasco, Alexandre R. M. Pelegrinelli, Mariana F. Silva, Laís F. Dela Bela, Felipe A. Moura, Jefferson Rosa Cardoso

**Affiliations:** 1 Laboratory of Biomechanics and Clinical Epidemiology, PAIFIT Research Group, Universidade Estadual de Londrina, Av. Robert Koch, 60, 86038-440 Londrina, PR Brazil; 2 Universidade Estadual do Centro-Oeste, Guarapuava, PR Brazil; 3 Laboratory of Applied Biomechanics, Universidade Estadual de Londrina, PR Londrina, Brazil; 4 Universidade Positivo, Curitiba, PR Brazil

**Keywords:** Isokinetic dynamometer, Torque, 3D surface map, Foot

## Abstract

**Background:**

Maintenance of the medial longitudinal arch (MLA) of the foot is fundamental during functional tasks and disorders can lead to clinical alterations. Studies have demonstrated that deficits in ankle isokinetic performance can predispose an individual to lower limb injuries.

**Objectives:**

To evaluate the muscular performance of cavus, planus, and normal feet by means of torque/body mass and the isokinetic phases, to generate 3D surface map analysis, and to verify whether there is a relationship between MLA height and arch height flexibility with isokinetic performance.

**Methods:**

The sample consisted of 105 healthy adult women, divided into three groups: normal, cavus, and planus. Assessment in concentric mode at 30, 60, and 90 °/s in the dorsiflexion and plantarflexion of the ankle joint were analyzed during the three isokinetic phases (acceleration, sustained velocity, and deceleration). The variables total range of motion, peak of torque (PT), and angle of PT were extracted within the sustained velocity.

**Results:**

In dorsiflexion at 60 °/s, the phase where the velocicty is sustained (load range phase) was higher in the planus group (M_ean_D_ifference_=10.9 %; ω^2^_p_ = 0.06) when compared with the cavus group. Deficits in the peak torque/body mass in dorsiflexion at 60 °/s (cavus feet: MD=-3 N.m/kg; ω^2^_p_ = 0.06; and planus feet: MD=-1.1 N.m/kg; ω^2^_p_ = 0.06) were also observed as well as in the 3D surface maps, when compared with the normal group. The flexibility of MLA had a negative correlation of PT at 30 °/s in cavus group. The heigth of MLA had a postive correlation with the PT for the cavus and planus group ate 60 °/s. All other results did not show differences between the groups.

**Conclusions:**

The planus groups showed a better capacity of attain and sustained the velocity in dorsiflexion in relation the cavus group. The cavus and planus group had deficts in torque in relation the normal. The correlations were weak between the measures of MLA and PT. Thereby, in general the differences between foot types showed small effect in isokinetic muscle performance measures of the plantar and dorsi flexores.

**Trial registration:**

Study design was approved by the IRB (#90238618.8.0000.5231).

## Background

Studies suggest that a relationship between the medial longitudinal arch of the foot (MLA) and the biomechanical alterations of the lower limb may predispose a person to pain and injuries such as lateral ankle sprain [1, 2]. The height of the MLA can be categorized as high (pes cavus), normal, or flat arch (pes planus) and clinically, the MLA height index has proven to be a robust measure [3–5] and disfunctions are often associated with foot type [6, 7].

Two studies support the fact that lower MLA flexibility may accompany higher stress in soft-tissue and medial injuries [8, 9]. In pes cavus, there is a shortening of soft tissues that support the longitudinal arch, such as the plantar fascia and tibialis posterior muscles, with common ankle instability and lateral foot overload [10, 11], leading to abnormal stress in the medial capsule-ligament structures of the foot [12].

MLA structure is linked to intrinsic factors such as sex, and women may be at a greater risk for soft-tissue injuries than males and may even have a posterior tibial tendon dysfunction, a condition that is associated with flatfoot [13, 14]. Women also have less flexibility in the MLA when compared to men [9], accompanied by greater stress in the MLA, increasing the risk of injuries such as plantar fasciitis [8]. Furthermore, the literature suggests that there are differences in muscle strength and patterns of muscle recruitment strategies between men and women [15–17]. Previous research has focused on the isometric and concentric analysis of ankle muscle performance only in men and compensations were identified in plantarflexion at 30 °/s with greater stress in the medial compartment of the ankle [18, 19]. Beside this, variations in the height of the MLA in men correlate negatively with peak torque per body mass (PT/BM) for cavus feet in plantarflexion at 120 °/s, when compared with other types of feet [19].

The isokinetic dynamometer is the gold standard for assessing ankle isokinetic performance [20]. Identifying deficits and their relationship with changes in the MLA of the foot height can be used clinically to identify an increased risk of predisposition to lower limb injuries and can be a marker of recovery during musculoskeletal treatment after injury or surgical interventions [21, 22], as well as giving a general indication of the functional capacity of a subject [20, 23]. The use of isokinetic devices involves three phases of range of motion (ROM): (a) acceleration; (b) sustained velocity or load range, and (c) deceleration [21, 22]. The majority of studies do not take into account these three isokinetic phases, which may lead to measurement errors at high contractile velocities, since the subject may not benefit from the machine-offered resistance. Another way of assessing ankle isokinetic performance is through 3D surface maps. This method considers the torque–angle–velocity relationship, creating a behavioral map of these three variables throughout movement [24], adding information about the muscle action during exercise performance at different velocities. Thus, the aims of this study were to evaluate the muscular performance of cavus, planus, and normal feet by means of torque/body mass and the isokinetic phases, to generate 3D surface map analysis, and to verify whether there is a relationship between MLA height and arch height flexibility with isokinetic performance.

## Methods

### Study Design

This is a cross-sectional study with descriptive and analytical components.

### Participants

In total, 105 asymptomatic adult women, recruited through social media, aged between 20 and 40 years with no pain, discomfort, or disorders in the lower limbs in the previous 12 months. Women with a history of prior surgery to musculoskeletal structures (bones, joint structures, nerves, and fractures) and/or deformities in the lower limbs were excluded. Women with neurological, cardiovascular, or musculoskeletal dysfunction that prevented them from performing the evaluation, or pregnant were also excluded.

The sample size was calculated using the program G*Power 3.1.9.4 [25]. The *F* test was used to find differences in means between three independent groups, with an effect size of 0.60, based on a previous study [26], an acceptable margin of error of 5 % (α), and 80 % power. The necessary number of women for each group was 35. The signed informed consent was obtained from all women and the study design was approved by the IRB-Universidade Estadual de Londrina (#90238618.8.0000.5231).

The anthropometric measurements of the feet were taken only in the dominant limb. The dorsal height (measured at 50 % of the total foot length) and truncated foot length (the distance from the posterior heel to the first metatarsal head) were measured along the medial border of the foot [14]. The dominant member was defined by asking the individual: [27].

The arch height flexibility (AHF) was defined as the change in arch height (distance from the dorsal surface to the ground) from sitting to standing because of the change in load borne by the arch during these activities. The flexibility of the MLA was calculated by change the height of the arch (distance from the dorsal surface to land) between sitting and standing positions (cm), divided by 0.4 times the body mass (kg) and multiplied by 100 N [28]. The sample was categorized into three groups according to the Arch Height Index (AHI) classification: 1, cavus feet; 2, normal feet, and 3, planus feet [3, 4]. The validity and reliability of the AHI were previously established by Butler et al. [3].

### Protocol

Isokinetic performances of plantarflexion (gastrocnemius, plantaris, soleus, and fibularis longus) and dorsiflexion (tibialis anterior, extensor hallucis longus, and extensor digitorum longus) were measured using the Biodex System 4® Dynamometer (Biodex Medical System Inc., Shirley, NY), in concentric mode at 30, 60, and 90 °/s, with a sampling frequency of 100 Hz, per the standard Biodex recommendations for ankle strength assessment [29].

The study required a single visit, during which the demographic data were collected (height, weight, and age), and assessment of the AHI and arch height flexibility were carried out. Before the tests, the warm-up consisted of 10 min on a stationary ergometer at a target speed of 30 km/h [30] under the supervision of the examiner. After the warm-up, women performed the equipment familiarization, with two repetitions before the test at each velocity. This was their first experience with ankle isokinetic testing. Finally, the real testing was carried out. Similar procedures to the warm-up and familiarization were performed according to the study [31]. The ankle isokinetic protocol was characterized by a set of six repetitions at each velocity, performed in a simple draw, with a rest period of 90 s between sets.

The women were instructed not to perform high intensity running exercise on the day of the test, since studies have verified that strength loss can occur after running events [32, 33] which could affect the test result.

Previously the test, the ROM limits was standardized (40 ° plantarflexion and 20 ° dorsiflexion), with a total of 60 degrees, for all the evaluated individuals, similar range of motion to the study [34] who tested the reliability of ankle isokinetic performance. The women were seated on the dynamometer, and stabilized with belts around the trunk, pelvis, and thigh. The dynamometer set-up was as follows: orientation = 90 °, tilt = 0 ° (axis direction), seat orientation: 90 °, seatback tilt: 70 °, and knee flexion: 30 °; the axis passed through the body of the talus and lateral malleolus or just above the medial malleolus. The women were instructed to perform maximum effort during all repetitions, and verbal encouragement and visual feedback were provided. Tests were assessed by the same researcher with experience in performing dynamometer isokinetic testing/treatment. For reliability purposes, a coefficient of variation of less than 10 %, for each set, was considered acceptable, as described in Malina et al. [35].

### Data processing

The ankle isokinetic data were processed using MATLAB® (The MathWorks, Inc., Natick, USA) algorithms. The torque signal of each test was first filtered by a second-order low-pass Butterworth filter, with a cut-off frequency of 10 Hz. In the three velocities performed, with five repetitions each, the average values of each specified variable were withdrawn. The percentages of each isokinetic phase (acceleration - ACC, sustained velocity - load range - LR, and deceleration - DC) were calculated in relation to the total range of motion. The mean of total ROM in each repetition was calculated, because the limits of range of motion is not achieved at all repetitions. Peak torque/body mass (PT/BM) and angle of peak of torque were also calculated only during the load range phase [22]. The PT/BM results is presented in percentage in relation the body mass.

### Statistical analysis

For statistical analyses, the numerical variables were evaluated for the distribution of normality using the Shapiro–Wilk test. The results are presented as mean ($\overline{x}$) and standard deviation, except for the anthropometric characteristics, which are presented in median and quartiles (25–75 %) because they failed to meet the Gaussian distribution. Mean difference (MD) and 95 % confidence intervals (CI) were also calculated between groups. Anthropometric variables were compared at the beginning of the study using one-way ANOVA or Kruskal-Wallis. The three groups were compared for isokinetic variables by means of one-way ANOVA, followed by the Levene test to confirm the homogeneity of the variances. If the *F* test was statistically significant, we used the Post-hoc of Bonferroni. For the results that showed differences between the groups, the effect size was calculated (partial omega-squared, ω^2^_p_), which is an estimate of how much variation in response variables is explained by explanatory variables; ω^2^_p_ can have values between ± 1, zero indicates no effect. If the observed *F* is less than one, the ω^2^_p_ will be negative. The magnitude of the effect was considered as 0.01 small 0.06 moderate, and 0.15 large [36, 37].

Pearson correlation coefficient (95 % CI) was calculated to verify the relationship between the variable flexibility and the height of the MLA and peak of torque (dorsiflexion/plantarflexion) in the sustained velocity phase. Significance was stipulated at 5 % and all analyses were performed with SPSS version 25.0 (IBM SPSS®, Armonk, NY, USA).

## Results

Anthropometric characteristics and other variables are shown in (Table 1). Table 2 showed the results of the isokinetic phases and the ROM. In plantarflexion, statistically significant differences were observed in the percentage of deceleration at 60 °/s in the cavus group (MD = 1.7 %; ω^2^_p_ = 0.05), when compared with the normal group. The ROM was higher in the planus group at 90 °/s in relation the normal group (MD = 2.6 °; ω^2^_p_ = 0.09).

**Table 1:** Anthropometric characteristics of the sample by groups and classification of foot types

Variables	Normal (*n* = 35)	Cavus (*n* = 35)	Planus (*n* = 35)	*P*
**Age (years):**				
Md (25–75 %)	25 (23.95–28.04)	23 (23.29–27.27)	21 (21.6–24.7)	0.07
**BMI (kg/m**^2^**):**				
Md (25–75 %)	23.53 (22.17–24.19)	22.83 (22.39;24.35)	22.7 (21.6;23.7)	0.55
**Abdominal circumference (cm):**				
Md (25–75 %)	75 (72.9–80)	78 (75–80)	71 (68.5–73.4)	0.24
**AHI:**				
$\overline{x}$(SD) [95 % CI]	0.35 (0.02) [0.34;0.35]	0.38 (0.01) [0.38;0.38]	0.28 (0.01) [0.38;0.38]	0.001^a^
**AFH (m/kgf):**				
$\overline{x}$(SD) [95 % CI]	1.42 (0.89) [1.28;3.33] Neutral	1.22 (0.84) [0.93;1.51] Rigid	2.80 (1.47) [2.29;3.31] Very flexible	0.004^b^

Md: median; 25–75 %: quartiles; *BMI* body mass index; $\overline{x}$ mean; *SD* standard deviation; *AHI* arch height index (Normal AHI: 0.30 to 0.37, *Cavus AHI* ≥ 0.38, Planus AHI: ≤ 0.29 [4]); *AHF* arch height flexibility (very rigid < 0.9, rigid 0.9 to 1.3, neutral 1.3 to 1.6, flexible 1.6 to 2.0 and very flexible > 2.0 [16]); and m/kgf: meter/kilogram force. ^a^ difference between groups. ^b^ difference between the cavus and planus groups.

**Table 2:** Isokinetic phases represented by the percentage of range of motion

Ankle	Normal$\overline{x}$(SD) [95 % CI] (*n *= 35)				Cavus$\overline{x}$(SD) [95 % CI] (*n* = 35)				Planus$\overline{x}$(SD) [95 % CI] (*n* = 35)			
**Plantar** **flexion**	**ACC (%)**	**LR (%)**	**DC (%)**	**ROM (°)**	**ACC (%)**	**LR (%)**	**DC (%)**	**ROM (°)**	**ACC (%)**	**LR (%)**	**DC (%)**	**ROM (°)**
30 °/s	8.27 (4.19) [6.81; 9.71]	51.36 (20.52) [44.31;58.41]	40.36 (18.86) [33.88;46.84]	52.31 (6.37) [50.12;54.50]	10.15 (6.82) [7.81;12.50]	40.23 (21.44) [32.86;47.58]	49.92 (17.23) [44.00;55.84]	53.71 (5.60) [51.78;55.63]	9.56 (4.60) [7.98;11.14]	45.65 (20.01) [38.78;52.53]	44.73 (19.03) [38.19;51.27]	54.93 (5.38) [53.08;56.7]
60 °/s	17.39 (20.31) [10.41;24.37]	73.21 (20.69) [66.10;80.31]	9.18 (3.35) [8.03;10.33]	54.77 (5.36) [52.93;56.61]	19.76 (19.13) [13.19;26.33]	72.77 (19.48) [66,08;79.47]	**7.44 (1.13) [7.05;7.83]**^**a**^	55.82 (4.37) [54.32;57.33]	24.01 (21.24) [16.72;31.31]	68.93 (18.01) [62.74;75.12]	8.06 (3.02) [7.02; 9.10]	56.78 (4.86) [55.11;58.45]
90 °/s	36.25 (25.78) [27.40;45.11]	52.72 (27.54) [43.25;62.18]	10.16 (4.17) [8.73;11.60]	55.60 (4.08) [54.19; 57]	37.44 (27.17) [28.10;46.77**]**	50.59 (29.05) [40.61;60.57]	11.23 (8.89) [8.87;14.98]	56.97 (2.62) [56.07;57.87]	45.81 (25.17) [37.16;54.45]	41.44 (29.42) [31.33;51.54]	12.95 (13.08) [8.46; 17.45]	**58.26 (2.49)**^**a**^ **[57.40;59.11]**
**Dorsi-** **flexion**	**ACC (%)**	**LR (%)**	**DC (%)**	**ROM (°)**	**ACC (%)**	**LR (%)**	**DC (%)**	**ROM (°)**	**ACC (%)**	**LR (%)**	**DC (%)**	**ROM (°)**
30 °/s	2.85 (1.28) [2.41; 3.29]	90.94 (4.15) [89.51;92.36]	6.17 (4.05) [4.78;7.57]	52.55 (5.75) [50.58;54.53]	3.57 (3.16) [2.48; 4.66]	89.46 (5.72) [87.50;91.43]	6.88 (3.55) [5.66; 8.10]	52.75 (5.16) [50,98; 54,52]	3.00 (2.83) [2.02;3.97]	90.70 (3.90) [89.36;92.04]	6.37 (3.49) [5.17; 7.57]	53.93 (6.07) [51.84;56.01]
60 °/s	7.10 (2.05) [6.40; 7.81]	73.74 (18.07) [67.44;80.05]	19.20 (16.34) [13.13;25.27]	54.97 (4.69) [53.35;56.58]	7.48 (2.15) [6.75; 8.22]	68.24 (17.44) [62.25;74.24]	24.04 (16.97) [18.21;29.87]	55.65 (4.18) [54.21;57.08]	6.66 (1.84) [6.02;7.29]	**79.21 (8.70) [76.22;82.21]**^**b**^	**13.94 (8.85) [10.90;16.98]**^**b**^	56.47 (4.06) [55.08;57.87]
90 °/s	17.51 (14.00) [12.70;22.32]	29.39 (25.65) [20.57;38.20]	53.16 (25.80) [44.29;62.02]	54.90 (4.65) [53.30;56.50]	18.01 (13.29) [13.44;22.57]	38.16 (28.36) [28.42;47.90]	44.01 (23.80) [35.83;52.19]	56.58 (3.73) [55.81;57.67]	15.91 (4.55) [14.34;17.47]	35.20 (23.39) [27.16;43.23]	48.93 (23.44) [40.87;56.98]	**57.94 (2.93)** **[56.93;58.95]**^**a**^

$\overline{x}$: mean; *SD* standard deviation; *95 % CI* confidence interval; *%* percentage of each phase in relation to the total range of motion; *ACC* acceleration; *LR* load range; *DC* deceleration; *ROM* range of motion; and deg: degrees. ^a^ Significant difference compared to the normal group.^b^ Significant difference between planus group and cavus group.

In dorsiflexion at 60 °/s, for the planus group, the DC phase was lower (MD = 10.1 %; ω^2^_p_ = 0.05) when compared with the cavus group. This difference occurred because the load range increases in the planus group in relation to the cavus group (MD = 10.9 %; ω^2^_p_ = 0.06). The range of motion was higher in the planus group (MD = 3.0 %; ω^2^_p_ = 0.09) at 90 °/s in relation the normal group.

For PT/BM in dorsiflexion at 60 °/s, the cavus (MD = − 3 %; ω^2^_p_ = 0.06) and planus (MD = − 1.1 %; ω^2^_p_ = 0.06) groups presented a significant deficit when compared to the normal group (Table 3). The others velocities and in plantarflexion not showed differences between the groups.

**Table 3:** Comparison of normalized peak torque by body mass and peak torque angle at different velocities

Ankle	Normal$\overline{x}$(SD) [95 % CI]		Cavus$\overline{x}$(SD) [95 % CI]		Planus$\overline{x}$(SD) [95 % CI]	
**Plantar** **flexion**	**PT/BM** **(%)**	**AngPT** **(°)**	**PT/BM** **(%)**	**AngPT** **(°)**	**PT/BM** **(%)**	**AngPT** **(°)**
30 °/s	96.99 (29.70) [86.78;107.19]	22.54 (3.00) [21.51;23.57]	88.71 (31.35) [77.94;99.48]	23.45 (4.63) [21.86;25.05]	85.89 (32.09) [74.86;96.91]	21.21 (5.69) [19.25;23.16]
60 °/s	78.93 (28.05) [69.29;88.57]	24.82 (6.39) [22.62;27.02]	76.62 (23.47) [68.55;84.69]	26.83 (8.91) [23.76;29.89]	65.46 (25.21) [56.79;74.12]	28 (6.86) [25.64;30.36]
90 °/s	58.42 (25.11) [49.79;67.05]	32.08 (9.17) [28.93;35.23]	54.87 (27.57) [45.40;64.35]	32.88 (10.87) [29.15;36.62]	51.09 (26.67) [41.92;60.25]	35.74 (11.47) [31.80;39.68]
**Dorsi-flexion**	**PT/BM** **(%)**	**AngPT** **(°)**	**PT/BM** **(%)**	**AngPT** **(°)**	**PT/BM** **(%)**	**AngPT** **(°)**
30 °/s	29.79 (5.04) [28.06;31.53]	17.03 (4.36) [15.53;18.53]	27.59 (5.48) [25.71;29.48]	17.38 (5.46) [15.50;19.26]	31.31 (13.15) [26.79;35.83]	16.85 (4.73) [15.22;18.48]
60 °/s	26.81 (4.58) [25.24;28.38]	18.10 (5.53) [16.20;20]	**23.88 (4.68)** [22.27;25.49]*	17.73 (5.79) [15.74;19.72]	**25.70 (4.79)** [25.05;28.34]*	20.22 (8.60) [17.26;23.17]
90 °/s	21.63 (3.35) [20.47;22.78]	11.40 (6.25) [9.26;13.55]	21.04 (5.08) [19.29;22.78]	10.86 (3.67) [9.60;12.12]	21.78 (3.97) [20.41;23.14]	13.26 (8.80) [10.23;16.29]

$\overline{x}$: mean; *SD* standard deviation; *95 % CI* confidence interval; *°/s* degrees per second; *PT/BM* peak torque/body mass; *AngPT* angle of peak of torque; and %: percentage of the peak of torque in relation the body mass.*P ≤ .05 compared with normal group (ω^2^_p_ = 0.06 in both).

**Fig. 1:**
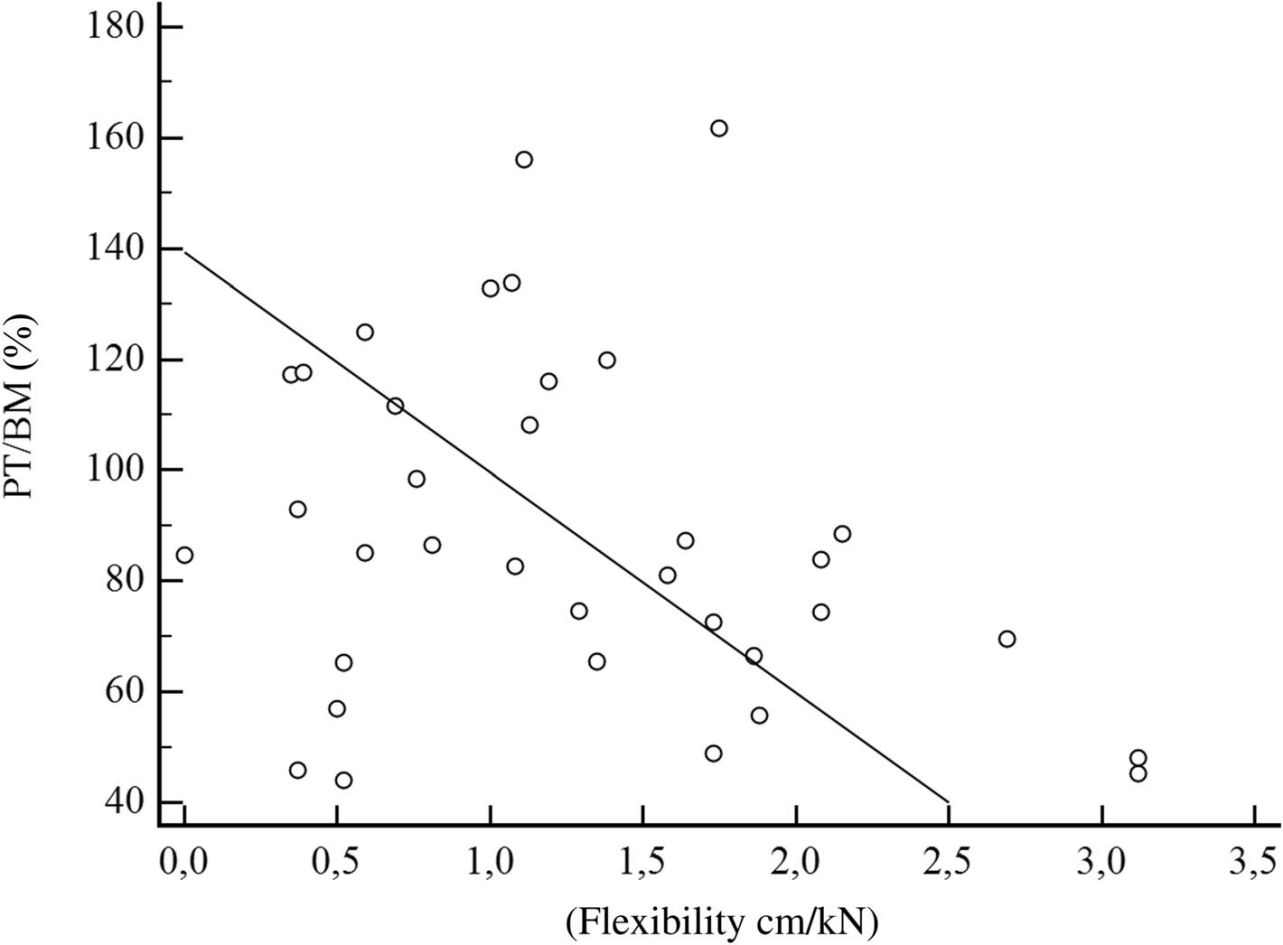
Scatter plot of the arch height flexibility with the peak torque/body mass (PT/BM) (cavus group) in 30 °/s plantarflexion

**Fig. 2:**
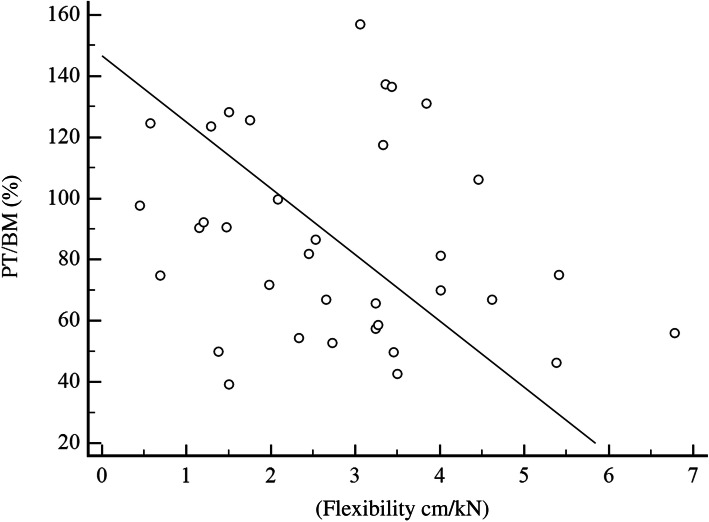
Scatter plot of the arch height flexibility with the peak torque/body mass (PT/BM) (planus group) in 30 °/s plantarflexion

In the correlation of the arch height flexibility with the PT/BM, the cavus group presented a significant negative result of *r* = − .37, 95 % CI [− 0.64; −0.01] (Fig. 1), where 14.3 % of the PT/BM variation at 30 °/s in plantarflexion may be credited with manipulating the value of the arch height flexibility. For the planus feet group, we observed that *r* = − .21, 95 % CI [− 0.50; 0.13] (Fig. 2), where 4.4 % of the variation of PT/BM at 30 °/s in plantarflexion can be credited with manipulating the value of the arch height flexibility.

Regarding the MLA height with PT/BM, the planus group presented a positive correlation of 0.36 95 % CI [0.03; 0.61]; that is, 12.9 % of the variability of PT/BM at 90 °/s in plantarflexion may be credited to the manipulation of the value of the MLA height index of the foot. In addition, *r* = .30 95 % CI [0.57; 0.03] was observed in the planus group, where 9 % of the PT/BM variability at 60 °/s in dorsiflexion could be explained by the variability of the MLA height index of the foot (Table 4).

**Table 4:** MLA height correlation with muscle performance (r)

	Normal (*n* = 35)	Cavus (*n* = 35)	Planus (*n* = 35)
**Plantar** **flexion**	**PT/BM (%)** $\overline{x}$**(SD);*r***	**PT/BM (%)** $\overline{x}$**(SD);*r***	**PT/BM (%)** $\overline{x}$**(SD);*r***
**30 °/s**	96.99 (29.70); − 0.01	88.71 (31.35); 0.04	85.89 (32.09); 0.25
**60 °/s**	78.93 (28.05); − 0.16	76.62 (23.47); − 0.07	65.46 (25.21); 0.25
**90 °/s**	58.42 (25.11); 0.10	54.87 (27.57); − 0.20	**51.09 (26.67); 0.36**
**Dorsi-flexion**	**PT/BM (%)** $\overline{x}$**(SD);*r***	**PT/BM (%)** $\overline{x}$**(SD);*r***	**PT/BM (%)** $\overline{x}$**(SD);*r***
**30 °/s**	29.79 (5.04); − 0.23	27.59 (5.48); 0.17	31.31 (13.15); 0.17
**60 °/s**	26.81 (4.58); − 0.30	23.88 (4.68); 0.07	**26.70 (4.79); 0.30**
**90 °/s**	21.63 (3.35); − 0.22	21.04 (5.08); 0.12	21.78 (3.97); 0.10

*MLA* Medial Longitudinal Arch; *r Pearson* correlation coefficient;$\overline{x}$: mean; *SD* standard deviation; *PT/BM* peak torque/body mass; and *%* percentage of the peak of torque in relation the body mass.

**Fig. 3:**
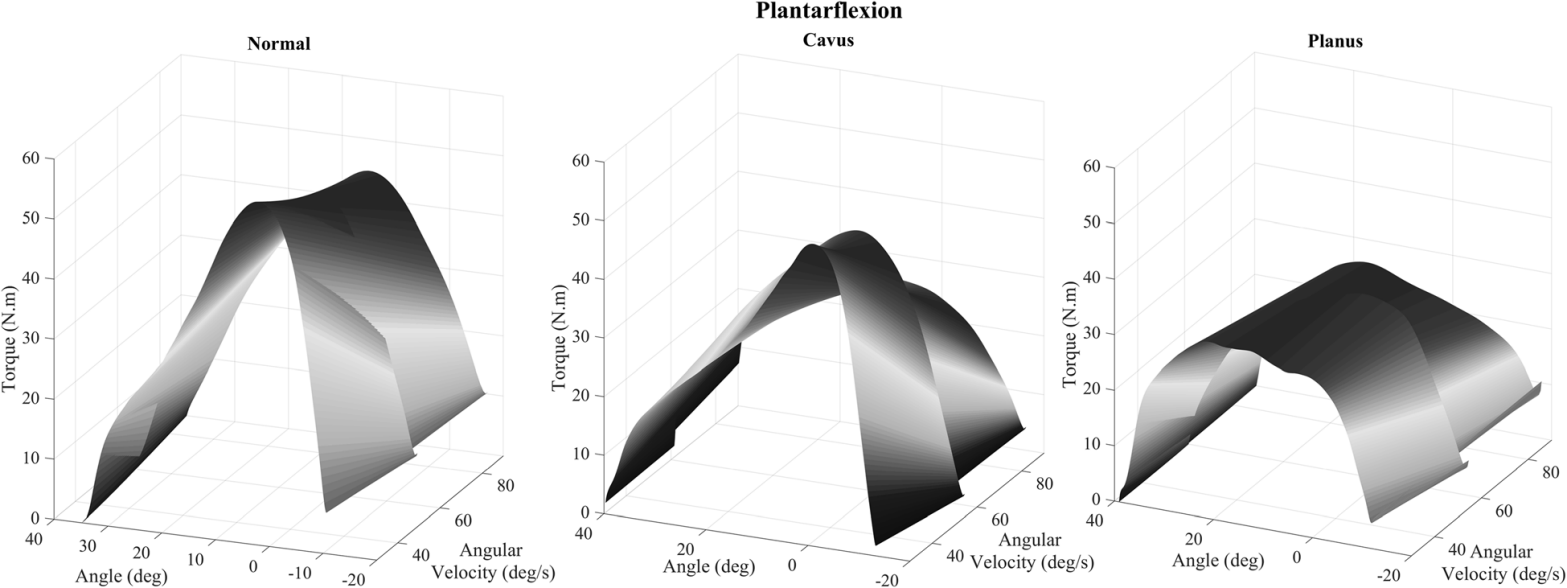
Surface map of the torque–angle–velocity relationship in plantarflexion; deg: degrees, N.m/kg: Newton meter/kilogram; and deg/s: degrees per second

**Fig. 4:**
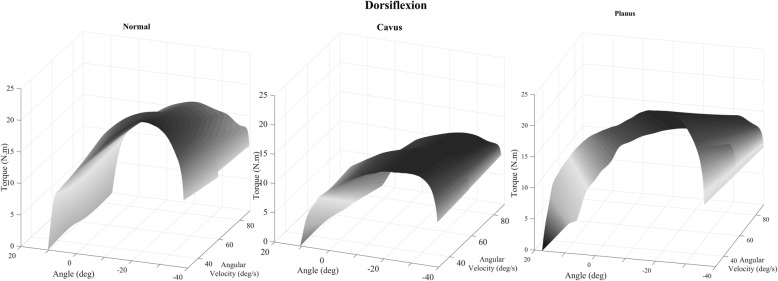
Surface map of the torque–angle–velocity relationship in dorsiflexion; deg: degrees; N.m/kg: Newton meter/kilogram; and deg/s: degrees per second

Mean peak torque and the resulting surface maps surface models are shown in (Figs. 3 and 4) for women for both plantarflexor and dorsiflexor muscles. The dark gray area indicates the moments in which there were higher values of the peak torque while the areas in light gray are the instants of smaller values of this variable. There is a progressive and constant decrease in peak torque with an increase in velocity in plantarflexion for the normal foot group. However, in the cavus foot group there was an abrupt drop in the peak torque at 60 °/s. The planus foot group presented lower peak torque at all velocities when compared to the normal and cavus feet groups.

In dorsiflexion, there was a steep decline in the peak torque at 60 °/s in the three groups of feet. The normal foot group demonstrated a higher peak torque peak at 90 °/s when compared to the cavus and planus feet groups. The cavus foot group had the lowest peak torque at 90 °/s when compared to the normal and planus feet group. Finally, the planus foot group showed lower peak torque as it presented a light gray color, mainly at 30 °/s, when compared to the normal and cavus feet groups.

## Discussion

The primary results of this study found that adult women with planus feet presented better capacity to attain and sustain the velocity (load range) in dorsiflexion at 60 °/s when compared with the cavus feet group. The torque showed differences only in dorsiflexion at 60 °/s. The PT/BM variable in dorsiflexion at 60 °/s (planus and cavus) presented a deficit with significance, when compared with the normal group.

In the correlation of the arch height flexibility with the PT/BM, the planus group presented a negative result with the variation in the PT/BM at 30 °/s in plantarflexion. Regarding the MLA height with the PT/BM, the planus feet group presented a positive correlation with the PT/BM at 90 °/s in plantarflexion. For all others variables analyzed the type of foot not influenced the isokinetic muscle performance in the muscles involved in plantarflexion and dorsiflexion.

Studies have pointed out an association between planus feet and excessive tension of the triceps sural, fibular, and posterior tibial tendons [38, 39]. The present study found an increased percentage of load range in dorsiflexion at 60 °/s in the planus group, due to a possible adaptation of muscle performance in view of posterior tibial tendon dysfunctions and greater tension in the sural triceps.

A significant deficit in PT/BM (approximately 7.6 %, with small effect size) in dorsiflexion at 60 °/s for the cavus and planus groups compared with the normal group was also observed in this study. It is possible these results are related, because planus feet lead to deformity of the forefoot associated with muscular imbalance of the leg, with weakness of the anterior tibial, and extensors of the toes [40, 41].

Individuals with planus feet are typically considered as having flexible feet, while individuals with cavus feet are more likely to have more rigid feet [19]. Planus feet in adults showed posterior tibial tendon insufficiency, resulting in significant deformity and disability [42]. Data that may support the results of the current study found that planus feet showed a statistically significant difference (8.2 %) in the load range at 60 °/s in dorsiflexion, when compared to the normal group. Although in most cases planus feet show no symptoms such as pain and/or marked musculoskeletal disorders, in hypermobile (flexible) planus feet, there is shortening of the Achilles tendon, which could explain a greater load range through muscle compensation mechanisms to achieve an adequate dorsiflexion movement [43].

This study verified that the cavus and planus feet groups presented classifications of MLA height flexibility as rigid and very flexible, respectively [14]. These changes were reflected in the negative correlation between flexibility and PT/BM at 30 and 60 °/s in plantarflexion, mainly for the cavus group; 14.2 % of the variability in foot flexibility can be explained by the variability in the PT/BM (or vice versa) and 85.8 % is not explained. Variance in scores is due to other factors such as the severity of malalignment of cavovarus, the grade of forefoot in plantarflexion and pronation, and both intrinsic and extrinsic muscle imbalance [44] at both velocities, when compared with the normal group.

Another relevant result was the correlation of the height of the MLA with the PT/BM at 90 °/s in plantarflexion, for the planus feet group this presented 12.9 % of the variability in the PT/BM at 90 °/s may be explained by the variability in the height of the MLA (or vice versa) and 87.1 % (exclusive variance) is not explained. Variance in scores is due to other factors such as age-related posterior tibial tendon degeneration, peritendinous injections, recurring micro traumas, collagen diseases, vascular reasons, and accessory navicular bone [45]. In dorsiflexion at 60 °/s, 9 % of shared variance and 81 % of variance were observed exclusively for the planus group between the height of the MLA and the PT/BM. Outcomes of the present study show, mainly in dorsiflexion at 60 °/s velocity, that the normal group presented improved muscular performance than the other two groups, with a higher PT/BM and lower ROM of acceleration to reach the sustained velocity.

A decrease in the peak torque was observed with an increase in the angular velocity, as demonstrated in other studies [46, 47]. Surface maps provide a comprehensive understanding of dynamic behavior of a muscle compared to static assessments, due to focusing on length − tension and length − velocity relationships [24]. These results may allow for identification of muscular deficits and help during treatments for foot dysfunctions. Adult acquired flatfoot deformity or other dysfunctions are common disorders that typically affect middle-aged adults over 40 years of age and older women, resulting in foot pain, malalignment, and loss of function [48]. The sample of this research was constituted by adult women up to 40 years old with different types of feet, which explains some of the small differences found in the muscular performance.

Ankle strength can be used clinically as a marker of recovery during musculoskeletal treatment after injury or surgical interventions [49]. The results of the present study pointed out that dorsiflexor muscles in planus feet should be strengthened mainly at 60 °/s.

The study has some limitations, for example, we evaluated only women aged 20–40 years, however, the foot arch is a structure that changes with age [50] with lower foot circumference in women over 55 years of age compared to younger adults [51], which can influence the height of the MLA. Secondly, the inversion and eversion movements were not performed since most of the subjects reported difficulties or discomfort when generating maximal torque in the medio–lateral direction at higher velocities, as in the study of Gosonova et al. [34]. Lastly, we did not test muscle strength in the eccentric mode, considering that the higher tension forces generated with this type of contraction might have put our participants at risk for muscle-tendon injuries.

## Conclusions

The planus foot group differed in load range from the cavus group at 60 °/s in dorsiflexion. For PT/BM in dorsiflexion to 60 °/s, the cavus and planus groups presented a significant deficit when compared to the normal group. The flexibility of the MLA correlated negatively with the cavus group in plantarflexion at 30 °/s.

## Data Availability

All data can be made available from the corresponding author upon reasonable request.

## Abbreviations

MLA: Medial longitudinal arch
MD: Mean difference
ω^2^_p_: Partial omega squared
3D: Tridimensional
IRB: Institutional review board
PT/BM: Peak torque per body mass
LR: Load range
ROM: Range of motion
Md: Median
25–75 %: Quartiles
BMI: Body mass index
x¯: Mean
SD: Standard deviation
AHI: Arch height index
AHF: Arch height flexibility
AngPT: Angle of peak of torque
N.m/kg: Newton meter/kilogram
°/s: Degrees per second
*r*: Pearson correlation coefficient

## References

[1] Denyer JR, Hewitt NLA, Mitchell ACS (2013). Foot structure and muscle reaction time to a simulated ankle sprain. J Athl Train.

[2] Riskowski JL, Dufour AB, Hagedorn TJ, Hillstrom HJ, Casey VA, Hannan MT (2013). Associations of foot posture and function to lower extremity pain: results from a population-based foot study. Arthrit Care Res.

[3] Butler R, Hillstrom H, Song J, Richards C, Davis I (2008). Arch height index measurement system: Establishment of reliability and normative values. J Am Podiat Med Assn.

[4] Redmond AC, Crosbie J, Ouvrier RA (2006). Development and validation of a novel rating system for scoring standing foot posture: the Foot Posture Index. Clin Biomech.

[5] Williams DS, Davis I (2000). Measurements used to characterize the foot and the medial longitudinal arch: reliability and validity. Phys Ther.

[6] Levy JC, Mizel MS, Wilson LS, Fox W, McHale K, Taylor DC, Temple HT (2006). Incidence of foot and ankle injuries in West Point cadets with pes planus compared to the general cadet population. Foot Ankle Int.

[7] Naudi S, Dauplat G, Staquet V, Parent S, Mehdi N, Maynou C (2009). Anterior tarsectomy long-term results in adult pes cavus. Orthop Traumatol Surg Res.

[8] Scher D, Belmont P, Bear R, Mountcastle S, Orr J, Owens B (2009). The incidence of plantar fasciitis in the United States Military. J Bone Joint Surg Am.

[9] Zifchock R, Davis I, Hillstrom H, Song J (2006). The effect of gender, age, and lateral dominance on arch height and arch stiffness. Foot Ankle Int.

[10] Chilvers M, Manoli A (2008). II. The Subtle Cavus Foot and Association with Ankle Instability and Lateral Foot Overload. Foot Ankle Clin.

[11] Lowe W, Chaitow L. Foot, ankle, and lower leg. In: Orthopedic Massage (Second Edition), pp. 77–115, edited by Lowe W, Chaitow L, Mosby: Edinburgh. 2009.

[12] Imhauser CW, Siegler S, Abidi NA, Frankel DZ (2004). The effect of posterior tibialis tendon dysfunction on the plantar pressure characteristics and the kinematics of the arch and the hindfoot. Clin Biomech (Bristol Avon).

[13] Funk DA, Cass JR, Johnson KA (1986). Acquired adult flat foot secondary to posterior tibial-tendon pathology. J Bone Joint Surg Am.

[14] Zifchock RA, Theriot C, Hillstrom HJ, Song J, Neary M (2017). The relationship between arch height and arch flexibility: a proposed arch flexibility classification system for the description of multidimensional foot structure. J Am Podiatr Med Assoc.

[15] Callahan DM, Foulis SA, Kent-Braun JA (2009). Age-related fatigue resistance in the knee extensor muscles is specific to contraction mode. Muscle Nerve.

[16] Clark B, Manini T, Thé D, Doldo N, Ploutz-Snyder L (2003). Gender differences in skeletal muscle fatigability are related to contraction type and EMG spectral compression. J Appl Physiol.

[17] Kay D, Gibson A, Mitchell M, Lambert M, Noakes T (2001). Different neuromuscular recruitment patterns during eccentric, concentric and isometric contractions. J Electromyogr Kines.

[18] Yates B, White S (2004). The incidence and risk factors in the development of medial tibial stress syndrome among naval recruits. Am J Sports Med.

[19] Zhao X, Tsujimoto T, Kim B, Tanaka K (2017). Association of arch height with ankle muscle strength and physical performance in adult men. Biol Sport.

[20] Dvir Z. Isokinetics: muscle testing, interpretation, and clinical applications. Churchill Livingstone, Edinburgh; New York; 2004.

[21] Brown L, Whitehurst M, Findley B, Gilbert R, Groo D, Jimenez J (1998). Effect of repetitions and gender on acceleration range of motion during knee extension on an isokinetic device. J Strength Cond Res.

[22] Brown LE, Whitehurst M, Gilbert R, Buchalter DN (1995). The effect of velocity and gender on load range during knee extension and flexion exercise on an isokinetic device. J Orthop Sport Phys.

[23] Neville C, Flemister AS, Houck JR (2010). Deep posterior compartment strength and foot kinematics in subjects with stage II posterior tibial tendon dysfunction. Foot Ankle Int.

[24] Hussain SJ, Frey-Law L (2016). 3D strength surfaces for ankle plantar- and dorsi-flexion in healthy adults: an isometric and isokinetic dynamometry study. J Foot Ankle Res.

[25] Faul F, Erdfelder E, Lang AG, Buchner A (2007). G*Power 3: a flexible statistical power analysis program for the social, behavioral, and biomedical sciences. Behav Res Methods.

[26] Buldt AK, Levinger P, Murley GS, Menz HB, Nester CJ, Landorf KB (2015). Foot posture is associated with kinematics of the foot during gait: A comparison of normal, planus and cavus feet. Gait Posture.

[27] Rein S, Fabian T, Zwipp H, Mittag-Bonsch M, Weindel S (2010). Influence of age, body mass index and leg dominance on functional ankle stability. Foot Ankle Int.

[28] Mulligan EP, Cook PG (2013). Effect of plantar intrinsic muscle training on medial longitudinal arch morphology and dynamic function. Man Ther.

[29] Biodex MS. Biodex multi-joint system. Clinical resource manual. 1991.

[30] Pincivero DM, Gandaio CM, Ito Y (2003). Gender-specific knee extensor torque, flexor torque, and muscle fatigue responses during maximal effort contractions. Eur J Appl Physiol.

[31] Pelegrinelli ARM, Guenka LC, Dias JM, Dela Bela LF, Silva MF, Moura FA, Brown LE, Cardoso JR (2018). Isokinetic muscle performance after anterior cruciate ligament reconstruction: a case-control study. Int J Sports Phys Ther.

[32] Denadai B, Greco C, Tufik S, De Mello M (2007). Effects of high intensity running to fatigue on isokinetic muscular strength in endurance athletes. Isokinet Exerc Sci.

[33] Lepers R, Pousson ML, Maffiuletti NA, Martin A, Van Hoecke J (2000). The effects of a prolonged running exercise on strength characteristics. Int J Sports Med.

[34] Gonosova Z, Linduska P, Bizovska L, Svoboda Z. Reliability of ankle foot complex isokinetic strength assessment using the Isomed 2000 dynamometer. Medicina. 2018;54:43. 10.3390/medicina54030043 PMC6122110 30344274

[35] Malina RM. Variation in body composition associated with sex and ethnicity. Champaign: Human Kinetics: Human Body Composition; 2005.

[36] Keppel G. Design and analysis: A researcher’s handbook. 3rd ed. Englewood Cliffs: Prentice-Hall, Inc; 1991.

[37] Olejnik S, Algina J (2003). Generalized eta and omega squared statistics: measures of effect size for some common research designs. Psychol Methods.

[38] Queen RM, Mall NA, Hardaker WM, Nunley JA 2. Describing the medial longitudinal arch using footprint indices and a clinical grading system. Foot Ankle Int. 2007;28:456–62. nd . 10.3113/FAI.2007.0456 17475140

[39] Yalcin E, Kurtaran A, Selçuk B, Onder B, Yildirim MO, Akyüz M. Isokinetic measurements of ankle strength and proprioception in patients with flatfoot. Isokinetic Exerc Sci. 2012;20:167–71.

[40] Grice J, Willmott H, Taylor H (2016). Assessment and management of cavus foot deformity. Orthop Trauma.

[41] Ortiz C, Wagner E, Keller A. Cavovarus foot reconstruction. Foot Ankle Clin. 2009;14:471–87. 10.1016/j.fcl.2009.03.006 19712886

[42] Giza E, Cush G, Schon LC. The flexible flatfoot in the adult. Foot Ankle Clin. 2007;12:251–71. vi. 10.1016/j.fcl.2007.03.008 17561199

[43] Erol K, Karahan AY, Kerimoğlu Ü, Ordahan B, Tekin L, Şahin M, Kaydok E (2015). An important cause of pes planus: the posterior tibial tendon dysfunction. Clin Pract.

[44] Maynou C, Szymanski C, Thiounn A (2017). The adult cavus foot. EFORT open reviews.

[45] Uzunca K (2010). Pathologies of foot arches and their clinical effects. J PM&R.

[46] Anderson DE, Madigan ML, Nussbaum MA (2007). Maximum voluntary joint torque as a function of joint angle and angular velocity: model development and application to the lower limb. J Biomech.

[47] Khalaf K, Parnianpour M, Karakostas T (2000). Surface response of maximum isokinetic ankle torque generation capacity. J Appl Biomech.

[48] Flores DV, Mejía Gómez C, Fernández Hernando M, Davis MA, Pathria MN. Adult Acquired Flatfoot Deformity: Anatomy, Biomechanics, Staging, and Imaging Findings. RadioGraphics 2019;39:1437–1460. 10.1148/rg.2019190046 31498747

[49] Olsson N, Karlsson J, Eriksson BI, Brorsson A, Lundberg M, Silbernagel KG (2014). Ability to perform a single heel-rise is significantly related to patient-reported outcome after Achilles tendon rupture. Scand J Med Sci Sports.

[50] Staheli LT, Chew DE, Corbett M (1987). The longitudinal arch. A survey of eight hundred and eighty-two feet in normal children and adults. J Bone Joint Surg Am.

[51] Tomassoni D, Traini E, Amenta F (2014). Gender and age related differences in foot morphology. Maturitas.

